# Parental Alcohol Intoxication, Adverse Childhood Experiences, and Negative Psychological Reactions to Childhood Adversities: Cross-Sectional and Prospective Data from the Population‑Based HUNT Study

**DOI:** 10.1007/s40653-024-00651-4

**Published:** 2024-08-13

**Authors:** Siri Håvås Haugland, Barbara Carvalho, Arve Strandheim, Tonje Holte Stea

**Affiliations:** 1 Department of Psychosocial Health, University of Agder, Grimstad, Norway; 2 Department of Health and Nursing Sciences, University of Agder, Kristiansand, Norway; 3 Department of Child and Adolescent Psychiatry, Trøndelag Hospital Trust, Levanger, Norway

**Keywords:** Trauma, Parental alcohol use, Adverse childhood experiences, Family, Adolescent

## Abstract

Children growing up with alcohol-dependent parents have elevated risk for adverse childhood experiences (ACEs), but few studies have assessed the adverse effects of occasional or frequent exposure to parental intoxication episodes. This study examined whether such exposure was associated with increased risk of ACEs and negative psychological reactions (NPRs) in adolescence and young adulthood. The study relied on cross-sectional and longitudinal data from the Trøndelag Health Study in Norway and included 2,230 adolescents (ages 13–19 years) followed up 11 years later. Self-report questionnaires were used to collect information about exposure to parental intoxication, ACEs, and NPRs in adolescence and NPRs in young adulthood. Seeing parents drunk occasionally was associated with increased odds of six ACEs (odds ratios 1.42 [95% confidence interval 1.17–1.73] to 2.08 [1.44–3.01]) and increased odds of one NPR in adolescence (1.46, 1.12–1.91) compared with those who had never seen their parents intoxicated. Compared with those who had never seen parents intoxicated, seeing parents intoxicated frequently was associated with increased odds of all ACEs measured (1.80 [1.00–3.23] to 3.27 [1.92–5.56]), two NPRs in adolescence (1.60 [1.02–2.50] and 2.06 [1.30–3.27]), one NPR in adulthood (3.56, 1.83–6.94), and the perception of childhood as difficult/very difficult (2.99, 1.51–5.93). In conclusion, exposure to intoxicated parents was associated with increased risk of ACEs and NPRs during childhood, even at low frequency. Frequent exposure to parental intoxication was also associated with NPR in young adulthood.

## Background

Adverse childhood experiences (ACEs) are a heterogenous group of potentially traumatic experiences in childhood that may substantially increase the risk for negative physical and mental health outcomes throughout life (Bellis et al., 2015, 2019; Ferrara et al., 2016; Hughes et al., 2017). It has been estimated that more than a quarter of cases of anxiety and depression among European adults can be attributed to ACEs (Bellis et al., 2019).

Adverse Childhood Experiences (ACEs) are measured using various approaches (Bellis et al., 2019; Hughes et al., 2017). However, the relationship of ACEs to adverse health outcomes and poor quality of life is consistent regardless of whether they are assessed with a single measure (Tomasdottir et al., 2015), more global measures (Vederhus et al., 2021), or the widely used 10-item instrument called the “Adverse Childhood Experiences questionnaire” developed by Felitti et al. (Felitti et al., 1998) more than 25 years ago.

A systematic review and meta-analysis (Bellis et al., 2019) reported a pooled prevalence of 23.5% individuals in the European population having experienced one ACE and 18.7% having experienced two or more ACEs. These findings indicate that a substantial portion of the population is potentially vulnerable to experiencing the negative consequences associated with ACEs, which represents a serious public health issue. Although ACEs are quite common (Bellis et al., 2019; Sethi et al., 2023), studies show that the risk of adverse health outcomes increases with multiple ACEs (Hughes et al., 2017). In their systematic review and meta-analysis, Petruccelli et al. (Petruccelli et al., 2019) found that exposure to multiple ACEs was associated with a range of negative health outcomes, including mental health problems. Compared with other health outcomes, negative psychosocial outcomes were even more likely with higher ACE scores.

Children who grow up in families with parental alcohol disorders or problems have an increased risk of experiencing ACEs, such as physical and emotional abuse, violence, neglect, household dysfunction, and parental separation (Anda et al., 2002; Haugland et al., 2021). Excessive alcohol use may increase the risk of harm to children both directly and indirectly. Excessive alcohol use may increase the risk of physical violence and neglective behavior that may directly lead to children experiencing adversities (Laslett et al., 2012). In addition, heavy alcohol consumption may impair parents’ ability to establish a safe environment for their children and this may increase the risk of adverse events for the children. For example, in certain situations when the children are in the presence of intoxicated parents, it may involve being around other adults who also engage in excessive alcohol consumption, which exposes the children to a potential risk of harm from adults other than their parents (Laslett et al., 2012). Indirectly, excessive alcohol use can diminish the capacity of parents to monitor and supervise their children, increasing the likelihood of adverse events occurring in settings beyond the family setting (Haugland et al., 2019).

However, there is a lack of comprehensive research based on general population data that has investigated the potential negative consequences of being exposed to episodes of parental intoxication. These episodes involve parents who may not meet the diagnostic criteria for an alcohol use disorder but engage in heavy alcohol consumption when in the presence of their children, occasionally or frequently. Nonetheless, even this level of alcohol use has been associated with detrimental outcomes in children and an increased likelihood of adolescent substance use (McGovern et al., 2020; Rossow et al., 2016; Haugland et al.,2019). In Norway, a significant number of adolescents, ranging from approximately 37–60%, have reported witnessing their parents being intoxicated or drunk on one or multiple occasions. (Haugland et al., 2015, 2019). The prevalence of exposure to parental intoxication is significantly higher compared to the prevalence of adolescents with parents whose drinking patterns align with clinically oriented screening measures, such as the CAGE instrument. For instance, previous identification using the CAGE instrument has revealed that 16% of teenage fathers and 5% of teenage mothers exhibit concerning drinking behaviors (Haugland et al., 2013).

Taking a socio-ecological perspective into account, it is important to recognize that the relationship between parental alcohol use and childhood adversities cannot be solely understood in terms of individual factors but should also include family-level and contextual factors such as socioeconomics and cultural norms surrounding alcohol consumption (Lloyd & Kepple, 2017). ACEs exhibit social patterns, with lower socioeconomic status being associated with an increased risk of various ACEs (Metzler et al., 2017). While the Nordic countries do not have the highest alcohol consumption rates in Europe, the drinking culture in these countries has historically been linked to heavy episodic drinking and intoxication (Kuntsche et al., 2004). From a public health perspective, it is imperative to gain a deeper understanding of the potential adverse effects associated with exposure to parental intoxication episodes, given that a large number of children are exposed to this.

A previous cross-sectional retrospective Norwegian study of the general population reported that a higher number of those exposed to parental alcohol problems also reported struggling with bad memories from childhood because of loss, betrayal, neglect, violence, or abuse compared with those who had not had this exposure (Haugland et al., 2021). Moreover, NPRs may re-trigger the trauma experienced in childhood, and develop cognitive bias that may increase the risk of mental health problems (Vrijsen et al., 2017).

ACEs and NPRs might represent a pathway between parental drinking and adverse outcomes for children, warranting more research that explores possible associations between these factors. Gaining a deeper understanding of the association between various patterns of parental alcohol intoxication and adverse childhood experiences (ACEs) is crucial for developing effective prevention programs, informing therapeutic interventions, and promoting resilience in vulnerable groups. This knowledge is essential in reducing ACEs and mitigating their lifelong effects on health, encompassing both short-term and long-term outcomes.

The aims of the present study were to examine possible associations between exposure to parental intoxication (occasionally or frequently) and ACEs, compare NPRs to ACEs between those with and without exposure to parental intoxication, and compare NPRs to childhood adversities and negative perception of childhood quality in young adulthood between those exposed and not exposed to parental intoxication.

Based on the existing evidence, we propose the following hypotheses:

1. Exposure to parental intoxication, both occasional and frequent, may be associated with an increased risk of adverse childhood experiences (ACEs). Furthermore, we expect the risk estimates to be higher among respondents who report frequent exposure to parental intoxication than among those who report only occasional exposure.
2. Among respondents who report at least one ACE, exposure to frequent parental intoxication will be associated with a higher risk of negative psychological reactions (NPR) compared to those who only report ACEs without frequent parental intoxication.
3. Respondents who report frequent exposure to parental intoxication will have an increased risk of long-term negative psychological reactions (NPR) compared to those without such exposure.

The literature examining the relationship between occasional parental intoxication and NPRs, as well as occasional parental intoxication and long-term NPRs, is scarce. Consequently, the analysis approach will be more exploratory in nature without predetermined specific hypotheses.

## Materials and Methods

### Sample

The present study was part of the Trøndelag Health Study (HUNT) in Norway, which is a comprehensive, population-based study conducted by the HUNT Research Centre (Faculty of Medicine and Health Sciences, Norwegian University of Science and Technology), Trøndelag County Council, Central Norway Regional Health Authority, and Norwegian Institute of Public Health.

In the period covering 2006 to 2008, all adolescents ages 13 to 19 years living in North-Trøndelag County in Norway were invited to participate in the Young-HUNT3, the third wave of the adolescent part of the HUNT study. A total of 8,200 respondents agreed to participate (78.4% response rate), and data were collected using a self-report questionnaire completed during school hours. This period is considered T1 for the current study.

Between 2017 and 2019, every adult living in North-Trøndelag County, Norway, was invited to participate in the HUNT4-N Survey, the fourth wave of the adult part of the HUNT study. A total of 56,042 respondents agreed to participate (54% response rate), and data were collected using a self-report questionnaire completed at home. This period was considered as T2 in the current study.

Participation in the HUNT studies was voluntary, and respondents were informed that they could withdraw from the studies at any time (Åsvold et al., 2023).

### Measurement Instruments

The questions, response categories, and definitions used in the survey are shown in Table 1.

**Table 1:** Young-HUNT3 (T1) and HUNT4 (T2): questions, response options and recoding of variables

Variable	Questions	Response options	Recoding
Variables at T1			
Seen parents intoxicated on alcohol	Have you ever seen either of your parents intoxicated?	1.Never 2.A few times 3.A few times a year 4.A few times a month 5.A few times a week	Recoded: 1 = 0 (never; ref) 2 or 3 = 1 (occasional) 4 or 5 = 2 (frequent)
	Have you ever experienced the following events:	1. No 2. Yes, the last year 3. Yes, during life	Recoded: 1 = 0 (have not had the experience; ref) 2 or 3 = 1 (have had the experience) * Combined two items that measured (1) experiences with peers and (2) experiences with adults. Due to low numbers, these were combined into “unpleasant sexual experiences.” For descriptive analyses, the score on each ACE was summed.
Experiences of adverse and potentially traumatic experiences (T1)	1. Death of a loved one		
	2. A catastrophe (fire, avalanche, tidal wave, hurricane, etc.)		
	3. Experienced violence (beaten or injured)		
	4. Witnessed violence to others		
	5. Unpleasant sexual experiences*		
	6. Other very terrifying, dangerous, or violent experiences		
	7. Severe accident		
	8. Painful and scary treatment at a hospital		
	9. Threats or physical bullying by peers at school		
NPRs after the event/experience (T1)	If you have experienced any of the above, do you still think a lot about what happened?	Yes/no	No = 0 (no NPR) (ref) Yes = 1 (NPR) Comment: These questions were given only to those who reported at least one ACE.
	If yes, do you have scary thoughts, imagine pictures, or hear sounds from what happened, even if you do not want to?	Yes/no	
	When anything reminds you of what happened, do you get very upset, scared, or sad?	Yes/no	
	Do you avoid talking about it, thinking about it, or having feelings about what happened?	Yes/no	
Socioeconomic status (familial financial situation)	How well off do you think your family is compared with most others?	1. About the same as most others 2. Better financial situation 3. Worse financial situation	Recoded: 1 and 2 = 0 (good financial situation; ref) 3 = 1 (poor financial situation)
Age at T1	Retrieved through population registries	Continuous variable	
Variables at T2			
Memories of childhood quality	Do you struggle with bad memories from childhood because of loss, betrayal, neglect, violence, or abuse?	1. To a very high degree 2. To a high degree 3. To a small degree 4. To a very small degree 5. Not at all	Recoded: 1–2 = 1 (very troubled by painful childhood memories) 3–7 = 0 (not very troubled by painful childhood memories; ref)
	When you think about your childhood, would you describe it as:	1. Very good 2. Good 3. Average 4. Difficult 5. Very difficult	Recoded: 1–3 = 0 (not difficult childhood; ref) 4–5 = 1 (difficult childhood)

### Explanatory Variable

#### Parental Alcohol Intoxication

Information about the frequency of exposure to parental alcohol intoxication was measured by a single item at T1: “Have you ever seen your parents intoxicated (drunk)?” The response options include ‘Never, ' ‘A few times a year, ' ‘A few times a month, ' and ‘A few times a week. ' The intervals between each level of the variable were not evenly distributed, making it unsuitable for treating as a continuous variable. We have recoded the response options ‘sometimes’ and ‘sometimes a year’ to be ‘occasional’ as these response options may be difficult to understand as very distinct. Furthermore, due to a smaller number of respondents in the ‘a few times a week’ category, we merged it with the ‘a few times a month’ category into a new ‘frequently’ category to ensure sufficient statistical power for this group. ‘Never’ was used as the reference category in multivariable analyses.

### Outcome Variables

#### ACEs

Nine ACE items measuring intrapersonal violence and lifetime trauma at T1 were derived from the Young-HUNT brief lifetime trauma screen, which was adapted from the UCLA PTSD Reaction Index (Steinberg et al., 2004). The original sexual abuse question was made less specific to meet research ethics board requirements, and some other adaptations to the Norwegian context were applied (e.g., a question on neighborhood shooting was omitted). The events include the death of a loved one, a catastrophe (such as a fire or hurricane), violence (being beaten or injured), witnessing violence to others, unpleasant sexual experiences, other very terrifying, dangerous, or violent experiences, severe accidents, painful and scary treatment at a hospital, and threats or physical bullying by peers at school. The response options were recoded into two categories: 0 (have not had the experience) and 1 (have had the experience). The items were intentionally maintained thematically, following the structure of the original questionnaire, rather than being combined into a single index. This decision was made to facilitate a more comprehensive exploration of the specific types of challenging childhood experiences that could be associated with parental alcohol intoxication. Our focus was on gaining a detailed understanding of the nuanced relationship between parental intoxication and various difficult childhood experiences, beyond solely examining its influence on the overall variation in the number of ACEs. For descriptive analyses, the response on each ACE was summed.

### NPRs

Those who had reported at least one ACE at T1 were instructed to respond to four items reflecting NPRs after the adverse event/experience, with response options yes/no on each of the four items. These items were based on the UCLA PTSD index (Steinberg et al., 2013). Specifically, they were asked if they still thought a lot about what happened, if they had scary thoughts, imagined pictures, or heard sounds from the event(s) even when they did not want to, if they felt very upset, scared, or sad when reminded of what happened, and if they avoided talking about it, thinking about it, or having feelings about what happened. Those who had no ACEs were instructed to skip these questions about NPRs.

At T2, NPRs were measured by a single item reflecting memories of childhood adversities that assessed the respondents’ struggles with bad memories from childhood, specifically related to loss, betrayal, neglect, violence, or abuse. The response options include “To a very high degree,” “To a high degree,” “To a small degree,” and “To a very small degree.” For recoding purposes, the options were recoded into two categories: 0 (not very troubled by painful childhood memories) and 1 (very troubled by painful childhood memories).

In addition, we included an item of self-perception of childhood quality, where the respondents were asked to describe their childhood when they think about it. The response options include “Very good,” “Good,” “Average,” “Difficult,” and “Very difficult.” For recoding purposes, the options were recoded into two categories: 0 (not difficult childhood) and 1 (difficult childhood).

Both questions are part of the HUNT Difficult Childhood Questionnaire, which has been previously validated by Vederhus et al. (Vederhus et al., 2021).

### Control Variables

Adolescent age, sex, and parental financial situation, which was used as a proxy for socioeconomic status (SES), were considered as possible confounders. The importance of controlling for these variables has been confirmed by studies showing that higher SES is associated with higher alcohol consumption (Strand & Steiro, 2003), exposure to parental intoxication increases by age within similar populations (Haugland et al., 2015), and adolescent outcomes may vary by sex (Haugland et al., 2013).

Age was applied as a continuous variable based on age at T1 (Young-HUNT3). Age was computed as the number of days between birth (as registered by the Norwegian National Registry) and the date of participation in the study, divided by 365.2425 (the average number of days per year in the Gregorian calendar), rounded to one decimal.

The sex of each respondent was retrieved by asking at T1 whether respondents were male or female (reference category).

As noted, the family financial situation was used as a proxy for SES. This variable measures how the respondents perceive their family’s financial situation compared to others. The response options include “About the same as most others,” “Better financial situation,” and “Worse financial situation.” For recoding purposes, the options were recoded into two categories: 0 (good financial situation) and 1 (poor financial situation). The question was based on the HUNT data bank derived from the World Health Organization collaborative cross-national Health Behaviour in School-aged Children study in Europe.

### Data Analysis

**Fig. 1:**
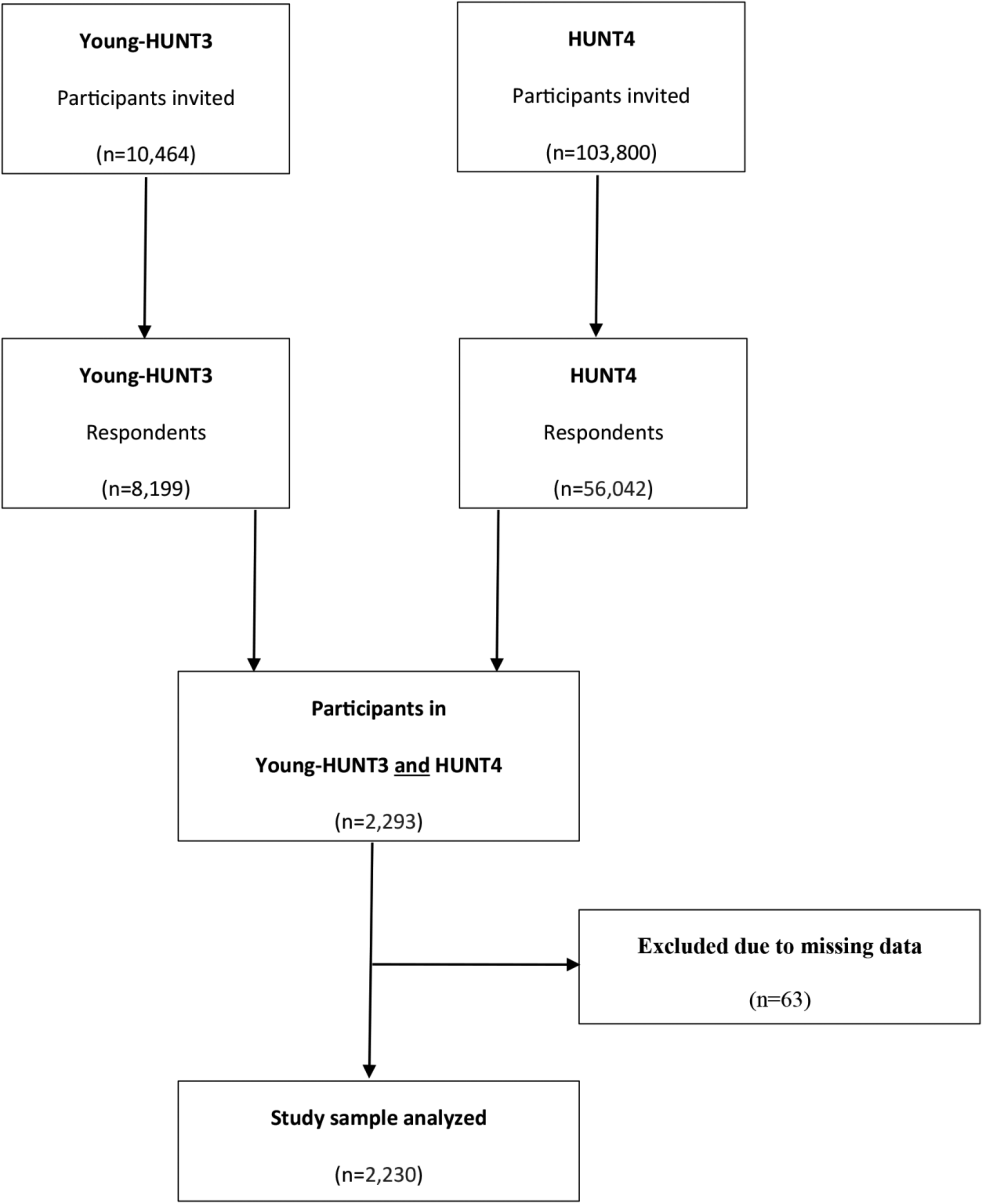
Flow chart of study population. HUNT: Trøndelag Health Study

Figure 1 provides a flow chart of the study population.

Initial descriptive analyses were performed to yield an overview of respondent characteristics (Table 2). Chi-squared tests were used to compare the prevalence of ACEs and/or NPRs between participants who had and had not been exposed to parental intoxication (Table 2). Multivariable logistic regression, adjusted for age, sex, and SES, was performed to investigate the association between exposure to parental intoxication and ACEs and or/NPRs.

Results were reported as odds ratios (ORs) with 95% confidence intervals (CIs). The level of statistical significance was set to 5%. All analyses were conducted using IBM SPSS Statistics 28.0.

## Results

### Descriptive Analysis

The present analysis applied cross-sectional and longitudinal data and included 2,230 adolescents (1,287 females and 943 males), ages 13–19 years, who participated in Young-HUNT3 (T1) and were followed up 11 years later in HUNT4 (T2) (Fig. 1).

Respondents had a mean age of 15.9 years (standard deviation [SD] 1.77) at T1. A total of 57.6% of the respondents were female, and 8.4% reported a poor financial situation in the family.

Among the respondents, 73.7% reported at least one ACE, 37.7% reported more than one, and 9.2% reported more than four ACEs. The mean number of ACEs for the total sample was 1.46 (SD 1.44). Mean ACE scores differed significantly between those exposed to parental alcohol intoxication (M = 1.67, SD = 0.042) and those who had not seen parents intoxicated (M = 1.13, SD = 0.41); t (2047) = -9.291, *p* ≤ 0.001, two-sample t-test).

An almost consistent pattern emerged in the initial cross-table, chi-square analysis (Table 2). The prevalence of the various ACEs and NPRs was higher with exposure to parental intoxication compared with no reported exposure.

**Table 2:** Prevalence of ACEs and NPRs in relation to level of exposure to parental alcohol intoxication

	Never exposed to parental intoxication	Occasional exposure to parental intoxication	Frequent exposure to parental intoxication	*P*
Death of a loved one	458 (56.1)	791 (67)	97 (66.4)	< 0.001
A catastrophe (fire, avalanche, tidal wave, hurricane, etc.)	47 (5.8)	131 (11.1)	17 (11.4)	< 0.001
Experienced violence (beaten or injured)	45 (5.5)	119 (10.1)	29 (19.6)	< 0.001
Witnessed violence to others	106 (13.0)	323 (27.3)	49 (32.9)	< 0.001
Unpleasant sexual experiences	35 (4.3)	68 (5.8)	14 (9.5)	0.032
Other very terrifying, dangerous, or violent experiences	180 (37.4)	331 (40.2)	52 (48.6)	n.s.
Severe accident	85 (10.4)	203 (17.3)	33 (22.4)	< 0.001
Painful and scary treatment at the hospital	44 (5.4)	88 (7.5)	17 (11.4)	0.018
Threats or physical bullying by peers at school	53 (6.5)	92 (7.8)	20 (13.4)	0.015
Prevalence of NPRs among those who reported experiences of at least one ACE described above				
If you have experienced any of the above,^a^do you still think a lot about what happened?	180 (37.4)	331 (40.2)	52 (48.6)	n.s.
If yes,^a^do you have scary thoughts, imagine pictures, or hear sounds from what happened, even if you do not want to?	59 (23.7)	125 (28.3)	28 (40.0)	0.026
When anything reminds you of what happened, do you get very upset, scared, or sad?^a^	137 (29.9)	294 (36)	48 (45.3)	0.003
Do you avoid talking about it, thinking about it, or having feelings about what happened?^a^	163 (35.6)	279 (34.8)	39 (37.5)	n.s.
Measures of NPR at T2, total sample				
Struggle with bad memories	35 (4.1)	57 (4.7)	17 (10.9)	< 0.001
Perceive childhood as difficult	33 (3.9)	45 (3.7)	16 (10.3)	< 0.001

P values were obtained using the Pearson chi-squared test. Data are presented as n (%) unless otherwise noted

n.s. = non-significant at the 0.05 level

^a^Only participants who reported at least one ACE answered this question

### Multivariable Logistic Regression Results

Multivariable logistic regression adjusted for sex, age, and SES (Table 3) showed that the odds of having experienced the death of a loved one increased if respondents had seen parents intoxicated occasionally (OR 1.42, CI 1.17–1.73) compared with never having seen parents intoxicated. The analysis further showed that those who had been exposed to intoxicated parents occasionally more often had experienced some kind of catastrophe (e.g., fire, avalanche, tidal wave, hurricane; OR 2.08, CI 1.44–3.01), as had those with exposure to intoxicated parents frequently (OR 2.18, CI 1.18–4.03). Experiences of violence were also more common with exposure to intoxicated parents occasionally (OR 1.60, CI 1.10–2.32) or frequently (OR 3.27, CI 1.92–5.56), as was having witnessed violence to others (occasionally: OR 1.91, CI 1.48–2.47; frequently: OR 2.38, CI 1.55–3.63). The odds of having other terrifying, dangerous, or violent experiences also increased for both frequencies of exposure (occasionally: OR 1.48, CI 1.11–1.97; frequently: OR 1.90, 1.18–3.05). Additionally, having been in a severe accident was more common with exposure to intoxicated parents either occasionally (OR 1.47, CI 1.11–1.96) or frequently (OR 1.95, CI 1.21–2.13). Compared with no exposure to intoxicated parents, those with frequent exposure more commonly had three additional types of ACEs: unpleasant sexual experiences (OR 2.01, CI 1.02–3.97), painful and scary treatment in a hospital (OR 2.15, CI 1.17–3.95), and being threatened or bullied physically in school (OR 1.80, CI 1.00–3.23).

**Table 3:** ACEs reported at T1 and exposure to parental intoxication

	Death of a loved one	A catastrophe	Experienced violence	Witnessed violence to others	Unpleasant sexual experiences	Other very terrifying, dangerous, or violent experience	Experienced a severe accident	Painful and scary treatment at a hospital	Threats or physical bullying by peers at school
Exposure to parental intoxication	OR (95% CI)	OR (95% CI)	OR (95% CI)	OR (95% CI)	OR (95% CI)	OR (95% CI)	OR (95% CI)	OR (95% CI)	OR (95% CI)
Occasionally	1.42 (1.17–1.73)***	2.08 (1.44–3.01)***	1.60 (1.10–2.32)*	1.91 (1.48–2.47)***	1.11 (0.71–1.73)	1.48 (1.11–1.97)**	1.47 (1.11–1.96)**	1.38 (0.94–2.04)	1.14 (0.78–1.65)
Frequent	1.39 (0.94–2.04)	2.18 (1.18–4.03)*	3.27 (1.92–5.56)***	2.38 (1.55–3.63)***	2.01 (1.02–3.97)*	1.90 (1.18–3.05)**	1.95 (1.21–2.13)**	2.15 (1.17–3.95)*	1.80 (1.00–3.23)*
(never; ref)									
Sex, male (ref. female)	0.74 (0.62–0.89)***	1.14 (0.84–1.54)	1.81 (1.33–2.46)***	1.97 (1.58–2.45)***	0.30 (0.18–0.48)***	0.93 (0.72–1.20)	0.84 (0.65–1.08)	1.11 (0.79–1.56)	1.15 (0.82–1.60)
Age at T1	1.11 (1.05–1.17)***	1.05 (0.96–1.15)	1.16 (1.16–1.27)***	1.32 (1.24–1.41)***	1.25 (1.12–1.40)***	1.15 (1.07–1.23)***	1.15 (1.07–1.24)**	1.02 (0.93–1.13)	1.08 (0.99–1.19)
Low SES at T1	1.06 (0.76–1.48)	0.61 (1.33–1.16)	1.95 (1.25–3.05)**	1.60 (1.12–2.29)**	0.30 (0.76–2.50)	2.03 (1.40–2.94)***	1.17 (0.77–1.78)	1.55 (0.92–2.59)	2.81 (1.82–4.34)***

* *p* < 0.05; **p < 0.010; ***p < 0.001

Respondents who had experienced at least one ACE were asked about psychological reactions in the time after the adverse event, and we compared the odds of these reactions between those who had and had not seen parents intoxicated. As Table 4 displays, getting very upset, scared, or sad when anything reminds them of what happened was more common among those who had seen parents drunk occasionally (OR 1.46, CI 1.12–1.91) or frequently (OR 2.06, CI 1.30–3.27). Additionally, those who had been frequently exposed to parental intoxication had increased odds (OR 1.60, CI 1.02–2.50) of thinking a lot about what happened compared with those who had not seen parents drunk. Those who had experienced ACEs and been exposed to parental intoxication did not differ from those who had experienced ACEs without such exposure in having scary thoughts, imagining pictures, or hearing sounds from what happened, even if not wanting to, or avoiding talking about it, thinking about it, or having feelings about what happened.

**Table 4:** Associations between being exposed to parental intoxication and experiencing negative psychological reactions after the event in adolescence

	Do you still think a lot about what happened?	If yes, do you have scary thoughts, imagine pictures, or hear sounds from what happened, even if you do not want to?	When anything reminds you of what happened, do you get very upset, scared, or sad?	Do you avoid talking about it, thinking about it, or having feelings about what happened?
Exposure to parental intoxication	OR (95% CI)	OR (95% CI)	OR (95% CI)	OR (95% CI)
Occasionally	1.17 (0.91–1.50)	1.12 (0.77–0.64)	1.46 (1.12–1.91)**	1.06 (0.82–1.37)
Frequently	1.60 (1.02–2.50)*	1.67 (0.92–3.05)	2.06 (1.30–3.27)**	1.21 (0.76–1.92)
(never; ref)				
Sex, male (ref. female)	0.39 (0.31–0.49)***	0.39 (0.26–0.58)***	0.33 (0.26–0.43)***	0.51 (0.40–0.65)***
Age at T1	1.00 (0.94–1.07)	1.09 (1.00–1.20)	1.00 (0.93–1.07)	0.93 (0.87–1.0)*
Low SES at T1	1.20 (0.82–1.75)	1.44 (0.87–2.38)	1.33 (0.90–1.96)	1.15 (0.78–1.70)

Based on cross-sectional analyses of data for those who reported at least one ACE at T1

* *p* < 0.05; **p < 0.010; ***p < 0.001

At T2, 11 years after T1, respondents were asked if they struggled with bad memories from childhood because of loss, betrayal, neglect, violence, or abuse (Table 5). The odds of this experience were more than tripled (OR 3.56, CI 1.83–6.94) among those who had been frequently exposed to parental intoxication during childhood compared with those who had not been exposed, but those who had seen parents intoxicated occasionally did not differ from the unexposed group. Having seen parents intoxicated frequently was also linked to increased odds of perceiving childhood as difficult/very difficult compared with not having had that exposure (OR 2.99, CI 1.51–5.93), but that was not the case for those who had seen parents drunk occasionally.

**Table 5:** Exposure to parental intoxication in childhood (T1) and NPRs to ACEs and negative perception of childhood quality as young adults (T2)

	Struggle with bad memories	Perceive childhood as difficult
Exposure to parental intoxication	OR (95% CI)	OR (95% CI)
Occasionally	1.39 (0.87–2.23)	1.07 (0.64–1.78)
Frequently	3.56 (1.83–6.94)***	2.99 (1.51–5.93)**
(never; ref)		
Sex, male (ref. female)	0.29 (0.17–0.49)***	0.71 (0.45–1.13)
Age at T1	0.78 (0.68–0.88)***	0.91 (0.80–1.03)
Low SES at T1	3.01 (1.78–5.11)***	3.10 (1.79–5.37)***

* *p* < 0.05; **p < 0.010; ***p < 0.001

## Discussion

In the present study, we examined the associations between exposure to parental intoxication and experiences of ACEs during childhood. In line with Anda et al. (2002), our findings revealed an increased risk of most ACEs for those with exposure to parental intoxication, regardless of the frequency of exposure, compared with those not having had this exposure. Specifically, the odds of having experienced the death of a loved one, a catastrophe, violence, or other terrifying experiences, having witnessed violence to others, or having been in a severe accident were increased for those who had seen their parents intoxicated (occasionally or frequently) compared with those who had not. In addition, the mean number of ACEs was significantly higher among those who had seen parents intoxicated. Compared with those not exposed to parental intoxication, those who reported having seen parents intoxicated frequently also had higher odds of having had unpleasant sexual experiences, having had painful and scary treatment in a hospital, or having been threatened or bullied physically in school.

Other studies have shown associations between parental alcohol use and adverse child outcomes such as injuries, hospitalization, or sexually offensive or violent experiences (Haugland et al., 2019, 2020; McGovern et al., 2020). Based on our data, being exposed to intoxicated parents seems to be consistently associated with increased odds of experiencing ACEs, but our data do not allow determination of whether parents were directly involved in the reported adverse events or whether parental intoxication caused them.

However, several factors may potentially explain the relationship between parental intoxication and ACEs. The toxic effects of alcohol may reduce parental ability to create a safe environment where children can thrive and develop well (Miller et al., 1997), at least during heavy drinking episodes. Children also may find their parents emotionally distant, unpredictable, or changed while intoxicated, which can be an upsetting experience (Foster et al., 2017). Good parenting practices such as showing care and support and maintaining open communication may be impaired when parents are heavy drinkers, making them less engaged and attentive to their children’s needs (Kelley et al., 2011; Lang et al., 1999; Su et al., 2018). Being with intoxicated parents also sometimes may include the presence of other adults who drink excessively, which can expose children to harm from others (Laslett et al., 2012; Miller et al., 1997). For example, Laslett et al. (Laslett et al., 2012) found that 10% of serious violence episodes witnessed at home because of other people’s drinking involved a family friend, and 30% of such episodes involved relatives other than parents or siblings. Furthermore, children may be left unsupervised during such episodes (Laslett et al., 2012), and parents may pay less attention to their children and offer less parental guidance (Lang et al., 1999). Downs and Miller (Downs & Miller, 1998) found that problematic paternal alcohol use may lead to a lack of protection that could increase the risk of sexual abuse of daughters by other male family friends.

Some events such as death in the family or experiencing a catastrophe such as a fire may also be difficult to the parents, and even for some increase the risk of alcohol use. In this context, it becomes challenging to establish a clear causal chain. We cannot definitively determine whether parental intoxication increases the risk of catastrophic events or whether parental alcohol consumption is influenced by such events. In our study, we observed that individuals who reported occasional or frequent parental intoxication were more likely to have experienced some form of catastrophe, such as fires, avalanches, tidal waves, or hurricanes. However, it is worth to note that tidal waves and hurricanes are not common occurrences in the study region, and there are limited reports of injuries related to avalanches. Hence, while we cannot definitively ascertain respondents’ experiences, it is plausible that when reporting the occurrence of a catastrophe, they may be referring to fires. Multiple reviews have provided evidence that alcohol intoxication amplifies the likelihood of fires and fire-related injuries or fatalities (Turner et al., 2017; Bruck et al., 2011), and a study from the US highlighted the presence of an intoxicated person in the household as the most significant factor in predicting fatal fires (Runyan et al., 1992). Moreover, a study conducted in New Zealand estimated that 24% of individuals who perished in alcohol-related fires were not the responsible drinkers (Connor & Casswell, 2012). However, it is important to note that information specifically concerning the harm caused to children in these incidents is lacking. Nonetheless, in line with our findings, these results suggest that parental intoxication may indeed play a substantial role in increasing the risk of fires.

The present results further indicated that NPRs were more common among adolescents who had experienced at least one ACE and been exposed to parental intoxication compared with those who had ACEs but had not seen parents intoxicated. Regardless of how often respondents with ACEs had been exposed in adolescence to parental intoxication, they had an increased risk of getting very upset, scared, or sad if anything reminded them of the ACE-related episode compared with those who had at least one ACE but no exposure to parental intoxication. For those reporting frequent exposure to parental intoxication and at least one ACE, the odds “of thinking a lot about what happened” also were increased compared with those with at least one ACE but no parental intoxication exposure.

Our findings add new knowledge about the possible psychological consequences of children being exposed to parental intoxication, as similar previous studies among non-clinical populations have mainly focused on other outcomes such as adolescent substance use (Rossow et al., 2016). In line with our findings, Hall and Webster (Hall & Webster, 2002) reported that children of parents with alcohol problems were more likely to report higher levels of trauma symptoms than those who had experienced trauma without parental alcohol problems. This difference could be the result of less effective stress management strategies, insufficient resources to address distress, and more at-risk patterns of responses among those who grew up with parental alcohol problems.

We further found that among those who reported having seen parents intoxicated frequently, the odds of struggling with bad memories from childhood because of loss, betrayal, neglect, violence, or abuse were strongly increased 11 years later compared with those who had never seen parents intoxicated. This result adds to similar cross-sectional findings in another general population (Haugland et al., 2021). The odds of perceiving childhood as difficult were in the current study tripled among those who had seen parents intoxicated frequently often compared with those who had never seen their parents drunk, but we found no significant differences between those with no exposure and those who had seen parents intoxicated a few times/a few times a year. For respondents with more frequent exposure, NPRs may have lasted into adulthood for several reasons. Being with intoxicated parents frequently represents a heavier life burden because parental intoxication dominates the family life more, and occasions facilitating alcohol-related adversity may be more frequent. It is plausible that alcohol-dependent parents are represented within this group, which thus may have included parents who were even heavier drinkers; the definition of intoxication was subjective for each respondent, and we lacked information about how much parents drank. ACEs and parental alcohol use are associated with both mental and physical adverse outcomes, but parents with alcohol problems may not respond adequately to their children’s health problems (Cleaver et al., 2011).

Although ACEs may increase risk for mental health problems in adulthood, not all people with these experiences have NPRs lasting into adolescence and adulthood. They may manage them without professional help, by themselves or with their family support. Some might need psychological treatment, most often with good results.

Our findings indicate that children who are often exposed to parental intoxication are vulnerable to long-term NPRs related to childhood adversities. Children who grow up with parental alcohol problems may have been unable to develop effective strategies to manage stress, leaving them less resilient and more vulnerable to NPRs to ACEs (Hall & Webster, 2002). The capacity to cope with and overcome adversity can be inhibited by several contextual factors associated with parental alcohol use, such as the parent–child relationship, parenting, parentification, and family conflicts (Park & Schepp, 2015). Furthermore, children who grow up with parental alcohol problems more often lack social support compared with those who grow up without parental alcohol problems (Haugland et al., 2021).

Resilience can be promoted by protective factors at the individual level (e.g., self-regulation, self-esteem), the family level (e.g., emotional support, positive parent–child relationships, family climate), and the community level (e.g., social support, mentorship, neighborhood amenities) (Brown & Shillington, 2017; Heard-Garris et al., 2018; Park & Schepp, 2015). Several protective factors can be promoted and offer a potential focus for interventions among vulnerable groups (Heard-Garris et al., 2018; Wingo et al., 2010). As such, future research would benefit from further understanding the moderating factors and pathways through which parental alcohol problems and other ACEs relate to resilience and psychological outcomes among children.

The finding that even infrequent exposure to intoxicated parents heightens the risk of a child experiencing traumatic events and subsequent negative psychological reactions is noteworthy for clinicians. These findings have the potential to drive changes in both public mental health initiatives and mental health services for both children and adults. The findings from the present study underscore the importance of raising awareness about ACEs linked to parental intoxication within communities and clinical practice. This awareness is crucial for preventing and mitigating the effects of adversity. Due to the intergenerational challenge associated with effects of alcohol intoxication, it is essential to use a family-centered approach to support those experiencing frequent parental alcohol intoxication episodes. Moreover, addressing the issue may require the involvement of various community services and the implementation of multiple strategies aimed at improving parenting practices and positively impacting the physical and mental health, as well as the social development of all family members.

Increasing knowledge of the increased risk of ACEs associated with occasional parental alcohol intoxication in the general population may also contribute to build community awareness and build community resilience.

Further research should identify what factors that may strengthen the resilience of children and families. Given the prevalence of exposure to parental intoxication, it is imperative for research to explore potential public health measures that could effectively prevent excessive alcohol use in the presence of children.

### Strengths and Limitations

A strength of this study was its longitudinal design based on a large, representative population of Norwegian adolescents with a long follow-up period of 11 years. This design enabled a prospective examination of the associations between ACEs and having seen parents intoxicated and whether NPRs to childhood adversities were more common among those who had this exposure compared with those who had not.

A general limitation of HUNT4 is the low participation rate, although the rate is still acceptable by contemporary international standards, especially for the age groups in this analysis. Participation was lowest in the youngest and oldest age groups (Åsvold et al., 2023). There is also potential selection bias in this current study because only participants who attended both Young-HUNT3 and HUNT4 were included. Prior analyses showed that non-participants in HUNT studies tend to have lower SES, higher mortality, and a higher prevalence of several chronic diseases (Langhammer et al., 2012).

The study relied solely on self-reported measures, which are prone to recall bias. The timeline from the ACE(s) and T1 is unknown. Another study comparing prospective versus retrospective reports of ACEs identified no bias in the retrospective evaluation of ACEs (Hardt et al., 2010). Colman et al. (Colman et al., 2016), however, found that concurrent mental health factors could affect consistency in reporting ACEs and that bias may be relevant in the estimation of associations between childhood adversity and outcomes in adulthood related to mental health.

The missing data on the ACE variables ranged from 4.8–5.2%. The original outcome variables were originally presented in a binary format (no/yes), and our only adjustment was combining the two yes options: ‘in the last year’ and ‘during life. ' However, given that the variables were applied individually rather than as a composite score, imputing missing data was deemed inappropriate. It is important to note that missing data may not occur completely at random, as participants who have experienced traumatic events may hesitate to respond to sensitive questions that could evoke memories of their difficult experiences. This could potentially result in an underreporting of ACEs, which, in turn, may have an impact on the outcomes and findings of the study. It is also important to note that within the group reporting exposure to parental intoxication, there may be parents who meet the diagnostic criteria for alcohol disorders, most likely among those who frequently engage in intoxicating levels of drinking.

## Conclusion

Exposure to parental alcohol intoxication was related to several different types of ACEs, even with an infrequent pattern of exposure. Among respondents who had one or more ACEs, those who also had been exposed to parental intoxication had an increased risk for NPRs in adolescence compared with those reporting no parental intoxication exposure. Frequent exposure to parental intoxication during childhood was associated with long-term NPRs in young adulthood. These findings are highly important for developing effective prevention programs to provide ACE-informed health services, strengthen resilience of vulnerable groups, and reduce ACEs and their life-course effect on health.

## Data Availability

Researchers linked to a Norwegian research institution may request HUNT data access from the HUNT Research Centre [www.ntnu.edu/hunt] after securing project approval from the Regional Committee for Medical and Health Research Ethics. Non–Norwegian-affiliated researchers should collaborate with and submit an application via a Norwegian principal investigator. Details on the application process and data access conditions can be found at [www.ntnu.edu/hunt/data] (Åsvold et al., 2023).

## References

[CR1] Anda, R. F., Whitfield, C. L., Felitti, V. J., Chapman, D., Edwards, V. J., Dube, S. R., & Williamson, D. F. (2002). Adverse childhood experiences, alcoholic parents, and later risk of alcoholism and depression. *Psychiatric Services (Washington D C)*, *53*(8), 1001–1009. 10.1176/appi.ps.53.8.1001 12161676 10.1176/appi.ps.53.8.1001

[CR45] Åsvold, B. O., Langhammer, A., Rehn, T. A., Kjelvik, G., Grøntvedt, T. V., Sørgjerd, E. P., Fenstad, J. S., Heggland, J., Holmen, O., Stuifbergen, M. C., Vikjord, S. A. A., Brumpton, B. M., Skjellegrind, H. K., Thingstad, P., Sund, E. R., Selbæk, G., Mork, P. J., Rangul, V., Hveem, K., Næss, M., & Krokstad, S. (2023). Cohort Profile Update: The HUNT study, Norway. *International Journal of Epidemiology*, *52*(1), e80–e91. 10.1093/ije/dyac095 35578897 10.1093/ije/dyac095 PMC9908054

[CR3] Bellis, M. A., Hughes, K., Leckenby, N., Hardcastle, K. A., Perkins, C., & Lowey, H. (2015). Measuring mortality and the burden of adult disease associated with adverse childhood experiences in England: A national survey. *Journal of Public Health (Oxford England)*, *37*(3), 445–454. 10.1093/pubmed/fdu065 25174044 10.1093/pubmed/fdu065 PMC4552010

[CR2] Bellis, M. A., Hughes, K., Ford, K., Ramos Rodriguez, G., Sethi, D., & Passmore, J. (2019). Life course health consequences and associated annual costs of adverse childhood experiences across Europe and North America: A systematic review and meta-analysis. *The Lancet Public Health*, *4*(10), e517–e528. 10.1016/S2468-2667(19)30145-8 31492648 10.1016/S2468-2667(19)30145-8 PMC7098477

[CR4] Brown, S. M., & Shillington, A. M. (2017). Childhood adversity and the risk of substance use and delinquency: The role of protective adult relationships. *Child Abuse & Neglect*, *63*, 211–221. 10.1016/j.chiabu.2016.11.006 27884507 10.1016/j.chiabu.2016.11.006

[CR5] Bruck, D., Ball, M., & Thomas, I. R. (2011). Fire fatality and alcohol intake: Analysis of key risk factors. J. Stud. Alcohol Drugs. 2011;72:731–736. 10.15288/jsad.2011.72.731 10.15288/jsad.2011.72.731 21906500

[CR6] Cleaver, H., Unell, I., & Aldgate, J. (2011). *Children’s needs—parenting capacity. Child abuse: Parental mental illness, learning disability, substance misuse and domestic violence*. The Stationery Office.

[CR7] Colman, I., Kingsbury, M., Garad, Y., Zeng, Y., Naicker, K., Patten, S., Jones, P. B., Wild, T. C., & Thompson, A. H. (2016). Consistency in adult reporting of adverse childhood experiences. *Psychological Medicine*, *46*(3), 543–549. 10.1017/S0033291715002032 26511669 10.1017/S0033291715002032

[CR8] Connor, J., & Casswell, S. (2012). Alcohol-related harm to others in New Zealand: Evidence of the burden and gaps in knowledge. *The New Zealand Medical Journal (Online)*, *125*(1360), 11–27. 22932651

[CR9] Downs, W. R., & Miller, B. A. (1998). Relationships between experiences of parental violence during childhood and women’s psychiatric symptomatology. *Journal of Interpersonal Violence*, *13*(4), 438–455. 10.1177/088626098013004002 9650246

[CR10] Felitti, V. J., Anda, R. F., Nordenberg, D., Williamson, D. F., Spitz, A. M., Edwards, V., Koss, M. P., & Marks, J. S. (1998). Relationship of childhood abuse and household dysfunction to many of the leading causes of death in adults. The adverse childhood experiences (ACE) study. *American Journal of Preventive Medicine*, *14*(4), 245–258. 10.1016/s0749-3797(98)00017-8 9635069 10.1016/s0749-3797(98)00017-8

[CR11] Ferrara, P., Guadagno, C., Sbordone, A., Amato, M., Spina, G., Perrone, G., Cutrona, C., Basile, M. C., Ianniello, F., Fabrizio, G. C., Pettoello-Mantovani, M., Verrotti, A., Villani, A., & Corsello, G. (2016). Child abuse and neglect and its psycho-physical and social consequences: A review of the literature. *Current Pediatric Reviews*, *12*(4), 301–310. 10.2174/1573396312666160914193357 27634538 10.2174/1573396312666160914193357

[CR12] Foster, J., Bryant, L., & Brown, K. (2017). *Like sugar for adults: The effect of non-dependent parental drinking on children & families*. Institute of Alcohol Studies.

[CR13] Hall, C. W., & Webster, R. E. (2002). Traumatic symptomatology characteristics of adult children of alcoholics. *Journal of Drug Education*, *32*(3), 195–211. 10.2190/U29W-LF3W-748L-A48M 12379051 10.2190/U29W-LF3W-748L-A48M

[CR14] Hardt, J., Vellaisamy, P., & Schoon, I. (2010). Sequelae of prospective versus retrospective reports of adverse childhood experiences. *Psychological Reports*, *107*(2), 425–440. 10.2466/02.04.09.10.16.21.PR0.107.5.425-440 21117468 10.2466/02.04.09.10.16.21.PR0.107.5.425-440

[CR19] Haugland, S. H., Holmen, T. L., Ravndal, E., & Bratberg, G. H. (2013). Parental alcohol misuse and hazardous drinking among offspring in a general teenage population: Gender-specific findings from the Young-HUNT 3 study. *Bmc Public Health*, *13*, 1140. 10.1186/1471-2458-13-1140 24314020 10.1186/1471-2458-13-1140 PMC3866523

[CR18] Haugland, S. H., Holmen, T. L., Krokstad, S., Sund, E. R., & Bratberg, G. H. (2015). Intergenerational hazardous alcohol use and area factors: The HUNT study, Norway. *Substance Use & Misuse*, *50*(14), 1753–1764. 10.3109/10826084.2015.1037396 26646627 10.3109/10826084.2015.1037396

[CR16] Haugland, S. H., Coombes, L., & Strandheim, A. (2019). Are sexually offensive or violent experiences more common among adolescents exposed to parental alcohol intoxication? *Child Abuse Review*, *28*(5), 366–380.

[CR17] Haugland, S. H., Coombes, L., & Strandheim, A. (2020). Parental alcohol intoxication and adverse health outcomes among offspring. A 4-year follow up HUNT study among 2399 Norwegian adolescents. *Preventive Medicine Reports*, *20*, 101170. 10.1016/j.pmedr.2020.101170 32817811 10.1016/j.pmedr.2020.101170 PMC7426560

[CR15] Haugland, S. H., Carvalho, B., Stea, T. H., Strandheim, A., & Vederhus, J. K. (2021). Associations between parental alcohol problems in childhood and adversities during childhood and later adulthood: A cross-sectional study of 28047 adults from the general population. *Substance Abuse Treatment Prevention and Policy*, *16*(1), 47. 10.1186/s13011-021-00384-9 34098987 10.1186/s13011-021-00384-9 PMC8186037

[CR20] Heard-Garris, N., Davis, M. M., Szilagyi, M., & Kan, K. (2018). Childhood adversity and parent perceptions of child resilience. *BMC Pediatrics*, *18*(1), 204. 10.1186/s12887-018-1170-3 29945566 10.1186/s12887-018-1170-3 PMC6020317

[CR21] Hughes, K., Bellis, M. A., Hardcastle, K. A., Sethi, D., Butchart, A., Mikton, C., Jones, L., & Dunne, M. P. (2017). The effect of multiple adverse childhood experiences on health: A systematic review and meta-analysis. *The Lancet Public Health*, *2*(8), e356–e366. 10.1016/S2468-2667(17)30118-4 29253477 10.1016/S2468-2667(17)30118-4

[CR22] Kelley, M. L., Pearson, M. R., Trinh, S., Klostermann, K., & Krakowski, K. (2011). Maternal and paternal alcoholism and depressive mood in college students: Parental relationships as mediators of ACOA-depressive mood link. *Addictive Behaviors*, *36*(7), 700–706. 10.1016/j.addbeh.2011.01.028 21392890 10.1016/j.addbeh.2011.01.028

[CR23] Kuntsche, E., Rehm, J., & Gmel, G. (2004). Characteristics of binge drinkers in Europe. *Social Science & Medicine*, *59*(1), 113–127. 10.1016/j.socscimed.2003.10.009 15087148 10.1016/j.socscimed.2003.10.009

[CR24] Lang, A. R., Pelham, W. E., Atkeson, B. M., & Murphy, D. A. (1999). Effects of alcohol intoxication on parenting behavior in interactions with child confederates exhibiting normal or deviant behaviors. *Journal of Abnormal Child Psychology*, *27*(3), 177–189. 10.1023/a:1021996122095 10438184 10.1023/a:1021996122095

[CR25] Langhammer, A., Krokstad, S., Romundstad, P., Heggland, J., & Holmen, J. (2012). The HUNT study: Participation is associated with survival and depends on socioeconomic status, diseases and symptoms. *BMC Medical Research Methodology*, *12*, 143. 10.1186/1471-2288-12-143 22978749 10.1186/1471-2288-12-143 PMC3512497

[CR26] Laslett, A. M., Ferris, J., Dietze, P., & Room, R. (2012). Social demography of alcohol-related harm to children in Australia. *Addiction (Abingdon England)*, *107*(6), 1082–1089. 10.1111/j.1360-0443.2012.03789.x 22229839 10.1111/j.1360-0443.2012.03789.x

[CR27] Lloyd, M. H., & Kepple, N. J. (2017). Unpacking the parallel effects of parental alcohol misuse and low income on risk of supervisory neglect. *Child Abuse & Neglect*, *69*, 72–84. 10.1016/j.chiabu.2017.03.007 28456067 10.1016/j.chiabu.2017.03.007 PMC5515626

[CR28] McGovern, R., Gilvarry, E., Addison, M., Alderson, H., Geijer-Simpson, E., Lingam, R., Smart, D., & Kaner, E. (2020). The association between adverse child health, psychological, educational and social outcomes, and nondependent parental substance: A rapid evidence assessment. *Trauma Violence & Abuse*, *21*(3), 470–483. 10.1177/1524838018772850 10.1177/1524838018772850 PMC7243080 29739281

[CR29] Metzler, M., Merrick, M. T., Klevens, J., Ports, K. A., & Ford, D. C. (2017). Adverse childhood experiences and life opportunities: Shifting the narrative. *Children and Youth Services Review*, *72*, 141–149. 10.1016/j.childyouth.2016.10.021 37961044 10.1016/j.childyouth.2016.10.021 PMC10642285

[CR30] Miller, B. A., Maguin, E., & Downs, W. R. (1997). Alcohol, drugs, and violence in children’s lives. *Recent Developments in Alcoholism: An Official Publication of the American Medical Society on Alcoholism the Research Society on Alcoholism and the National Council on Alcoholism*, *13*, 357–385. 10.1007/0-306-47141-8_19 9122502 10.1007/0-306-47141-8_19

[CR31] Park, S., & Schepp, K. G. (2015). A systematic review of research on children of alcoholics: Their inherent resilience and vulnerability. *Journal of Child and Family Studies*, *24*(5), 1222–1231. 10.1007/s10826-014-9930-7

[CR32] Petruccelli, K., Davis, J., & Berman, T. (2019). Adverse childhood experiences and associated health outcomes: A systematic review and meta-analysis. *Child Abuse & Neglect*, *97*, 104127. 10.1016/j.chiabu.2019.104127 31454589 10.1016/j.chiabu.2019.104127

[CR33] Rossow, I., Felix, L., Keating, P., & McCambridge, J. (2016). Parental drinking and adverse outcomes in children: A scoping review of cohort studies. *Drug and Alcohol Review*, *35*(4), 397–405. 10.1111/dar.12319 26332090 10.1111/dar.12319 PMC4950034

[CR34] Runyan, C. W., Bangdiwala, S. I., Linzer, M. A., Sacks, J. J., & Butts, J. (1992). Risk Factors for Fatal Residential Fires New Engl. J. Med. 1992;327:859–863. 10.1056/NEJM199209173271207 10.1056/NEJM199209173271207 1508246

[CR35] Sethi, D., Yon, Y., Parekh, N., Anderson, T., Huber, J. (2023). (‎2018)‎. *European status report on preventing child maltreatment*. World Health Organization. Regional Office for Europe. Retrieved August 13, from https://apps.who.int/iris/handle/10665/342240

[CR36] Steinberg, A. M., Brymer, M. J., Decker, K. B., & Pynoos, R. S. (2004). The University of California at Los Angeles post-traumatic stress disorder reaction index. *Current Psychiatry Reports*, *6*(2), 96–100. 10.1007/s11920-004-0048-2 15038911 10.1007/s11920-004-0048-2

[CR37] Steinberg, A. M., Brymer, M. J., Kim, S., Briggs, E. C., Ippen, C. G., Ostrowski, S. A., Gully, K. J., & Pynoos, R. S. (2013). Psychometric properties of the UCLA PTSD reaction index: Part I. *Journal of Traumatic Stress*, *26*(1), 1–9. 10.1002/jts.21780 23417873 10.1002/jts.21780

[CR38] Strand, B. H., & Steiro, A. (2003). Alkoholbruk, inntekt og utdanning i Norge 1993–2000 [Alcohol consumption, income and education in Norway, 1993–2000]. *Tidsskrift for den Norske Laegeforening: Tidsskrift for Praktisk Medicin ny Raekke*, *123*(20), 2849–2853. 14600708

[CR39] Su, J., Kuo, S. I., Aliev, F., Guy, M. C., Derlan, C. L., Edenberg, H. J., Nurnberger, J. I., Jr, Kramer, J. R., Bucholz, K. K., Salvatore, J. E., & Dick, D. M. (2018). Influence of parental alcohol dependence symptoms and parenting on adolescent risky drinking and conduct problems: A family systems perspective. *Alcoholism Clinical and Experimental Research*, *42*(9), 1783–1794. 10.1111/acer.13827 29969154 10.1111/acer.13827 PMC6120770

[CR40] Tomasdottir, M. O., Sigurdsson, J. A., Petursson, H., Kirkengen, A. L., Krokstad, S., McEwen, B., Hetlevik, I., & Getz, L. (2015). Self-reported childhood difficulties, adult multimorbidity and allostatic load. A cross-sectional analysis of the Norwegian HUNT study. *PloS One*, *10*(6), e0130591. 10.1371/journal.pone.0130591 26086816 10.1371/journal.pone.0130591 PMC4472345

[CR41] Turner, S. L., Johnson, R. D., Weightman, A., Rodgers, S., Arthur, G., Bailey, R., & Lyons, R. A. (2017). Risk factors associated with unintentional house fire incidents, injuries and deaths in high-income countries: A systematic review. Inj. Prev. 2017;23:131–137. 10.1136/injuryprev-2016-042174 10.1136/injuryprev-2016-042174 28119340

[CR42] Vederhus, J. K., Timko, C., & Haugland, S. H. (2021). Adverse childhood experiences and impact on quality of life in adulthood: Development and validation of a short difficult childhood questionnaire in a large population-based health survey. *Quality of Life Research: An International Journal of Quality of Life Aspects of Treatment Care and Rehabilitation*, *30*(6), 1769–1778. 10.1007/s11136-021-02761-0 33534031 10.1007/s11136-021-02761-0 PMC8178145

[CR43] Vrijsen, J. N., van Amen, C. T., Koekkoek, B., van Oostrom, I., Schene, A. H., & Tendolkar, I. (2017). Childhood trauma and negative memory bias as shared risk factors for psychopathology and comorbidity in a naturalistic psychiatric patient sample. Brain Behav. 2017; 7:e00693. 10.1002/brb3.693 10.1002/brb3.693 PMC5474701 28638703

[CR44] Wingo, A. P., Wrenn, G., Pelletier, T., Gutman, A. R., Bradley, B., & Ressler, K. J. (2010). Moderating effects of resilience on depression in individuals with a history of childhood abuse or trauma exposure. *Journal of Affective Disorders*, *126*(3), 411–414. 10.1016/j.jad.2010.04.009 20488545 10.1016/j.jad.2010.04.009 PMC3606050

